# Italian Physical Fitness Decline: A True Fact or a Mindset? A 10-Year Observational Perspective Study

**DOI:** 10.3390/ijerph17218008

**Published:** 2020-10-30

**Authors:** Nicola Lovecchio, Matteo Giuriato, Vittoria Carnevale Pellino, Francesca Valarani, Roberto Codella, Matteo Vandoni

**Affiliations:** 1 Laboratory of Adapted Motor Activity (LAMA), Department of Public Health, Experimental and Forensic Medicine, University of Pavia, 27100 Pavia, Italy; vittoria.carnevalepellino@unipv.it (V.C.P.); matteo.vandoni@unipv.it (M.V.); 2 Department of Human and Social Science, University of Bergamo, 24100 Bergamo, Italy; 3 Department of Biomedical Sciences for Health, Università degli Studi di Milano, 20133 Milano, Italy; francesca.valarani@gmail.com (F.V.); roberto.codella@unimi.it (R.C.); 4 Department of Human Science, University of Verona, 37100 Verona, Italy; matteo.giuriato@univr.it; 5 Department of Neuroscience, Biomedicine, and Movement Science, Università of Verona, 37100 Verona, Italy; 6 Department of Physical Education, Gdańsk Academy of Physical Education and Sport, 80-001 Gdańsk, Poland; 7 Department of Industrial Engineering, University of Tor Vergata, 00100 Rome, Italy; 8 Department of Endocrinology, Nutrition and Metabolic Diseases, IRCCS MultiMedica, 20138 Milano, Italy

**Keywords:** physical fitness decline, motor performance, youth performance, motor skills

## Abstract

Evidence regarding a putative physical fitness decline remains less well documented for Italian children and adolescents. An update review of data collection articles was undertaken concerning motor performances (power, strength, speed-agility and indirect cardiorespiratory fitness) of children and adolescents worldwide and compared with 2859 11–12-year-old Italian students of both sexes, throughout a ten-year observational period. Lower limb explosive strength (standing broad jump), flexibility (sit-and-reach), endurance (Cooper) and speed (SP-30) performances of sixth grade Italian students showed nearly stable trends, with no differences during the observed decade, in both sexes. This 10-year perspective study confirmed that Italian physical fitness levels flatlined rather than actually declined. According to these study data, the decline in physical fitness of the Italian youth is ostensible and needs a further in-depth analysis.

## 1. Introduction

A recession in physical fitness levels of young generations has been described as a consequence of growing industrialization [1] and has also been hyped by mass media [2]. The leading cause of this decline seems to be sedentary lifestyles [3], mainly associated with watching television/videogames, the use of motorized vehicles and diminished outdoor activities [4]. In particular, the loss of public spaces [5] and green areas caused by urbanization [6,7], as well as parents’ concerns in leaving children unsupervised [8] significantly reduced the opportunities to play outdoors [7]. Several factors affect adherence to physical activity, such as socio-economic status [9], growth phases [10], urban/rural context [11], availability of facilities [1] and parents’ cultural habits [12]. Altogether these result in the reduction of spontaneous motion and energy expenditure, contributing to the alarming increase of pediatric overweight and obesity [13]. A variety of studies showed critical decline in physical fitness levels of young populations over the years [12]. For instance, Tomkinson et al. [12] reported a decrease in aerobic capacity in developmental age (from 6 to 19 years) over 45 years (1958–2003) among twenty-seven European countries. Similarly, reductions in motor skills of Northern European countries (power, muscle strength and speed-agility) were highlighted by other authors [14,15]. On the contrary, other studies reported no difference in maximum rate of oxygen consumption (VO_2max_) in three different cohorts of Danish adolescents, assessed in 1983, 1997, 2003 [16], while two cohorts of 15-year-old females and males, assessed for VO_2max_ and jump height, in 1988 and 2001, revealed worst performance only in the respective lowest quartile [17]. Westerstahl et al. [18] found that Swedish 16-year-old boys had a better score in 1995 than in 1974 as to the performance of Sargent jump-and-reach test, particularly after body adjustments. Furthermore, Albon et al. [19] declared that it is not clear whether the entire New Zealand childhood population (10 to 14 years) is becoming less aerobically fit (from 1991 to 2001). In a sample of soldiers during entry recruitment, no relevant differences were found in male performances from 1975 to 2013, while females slightly improved VO_2_max. Moreover, muscular endurance (push-ups, sit-ups) revealed a progressive increase of strength over years [20]. Analogous data were collected during Swiss Armed Forces mandatory recruitment (2006–2015): performances in aerobic endurance [21] and muscle power (standing broad jump and medical shot ball) showed no variations over time [21]. Indeed, recently, Pinoniemi et al. [22], by comparing the broad jump performance in 65,527 United States youth (10–17 years old), between 1911 and 1990, registered a small increase of 12.6 cm (collectively considered), whereas 10–12-year-old children and adolescents (13–17 years old) kept constant results. Thus, previous studies are inconclusive about the entity of the physical fitness trend achieved by young populations. Likewise, there is a lack of valuable performance data in the Italian youth population. Not only an Italian trend is objectively lacking in the body of the scientific literature, but also the authors debate on a possible, unsubstantiated, physical fitness decline, and whether this might be an erroneous mindset, particularly when cardiorespiratory assessments are accounted. Our hypothesis is that the physical fitness trend is similar among Italian adolescents in these last decades. For this reason, the purpose of this perspective observational study was to determine the existence and the magnitude of a putative trend of physical fitness levels over a decade (2004–2013) in an Italian student population.

## 2. Materials and Methods

### 2.1. Participants

A total of 2859 students recruited from the same middle schools in North Italy were invited to participate in a cross-sectional study for ten years. Over the decade 2004–2013, at the beginning of the scholastic calendar, during the first 15 days of the scholastic course (second half of September in Italy) only sixth grade students (11 years old) were tested. The choice of the sixth grade students was due to the change of the scholastic curriculum following the primary course. The numerosity of the sample, by gender and school year, was reported in Table 1.

During two consecutive curricular physical education (PE) lessons from 8.00 to 13.00 a.m., children were invited by the same PE teacher to perform the same physical fitness test. Each class was composed of 18 to 27 children. As inclusion criteria, students were healthy and eligible to perform PE (as certified upon medical examination). Every parent/guardian was informed before study participation and signed an informed consent including the following information: name of the teacher, description of the procedures to be followed, description of any reasonable risks or discomforts (the study did not involve other kinds of risks except the general risks common to PE lessons), explanation of subject’s rights. Children provided verbal assent after a specification that the agreement in this survey was free and no extra academic credits were awarded for participation.

The study protocol was approved by the ethical boards of the school in accordance with the Declaration of Helsinki, as revised in 1983. All participants were free to withdraw their participation at any time. Written informed consent was obtained from the parents or legal guardians during the official enrollment while verbal assent was obtained from the children prior to participation.

### 2.2. Tests

The data collection consisted of a series of physical fitness tests selected within the widespread Eurofit Battery [23] and from usual practice considering the school settings conditions [23,24,25]. All data were collected by four sport science experts in full compliance and collaboration with the curricular PE teacher. These tests are reliable and valid instruments to measure physical fitness in children [25,26] and are strictly defined, free from operator’s influence, simple to administrate and cheap [27,28].

#### 2.2.1. Standing Broad Jump, SBJ

Participants were instructed to perform horizontal jump (systematic error nearly to 0) [29] trying to obtain the maximum distance. Each student started from a standing position placing both feet behind the starting line. After preparatory movements, a horizontal jump with free swing upper limb contribution was performed. The distance (to the nearest 0.5 cm) from the starting line to the heel of the rear-foot was recorded. The test was performed two times with a five minute rest between each attempt and the best score was retained for investigation.

#### 2.2.2. Sit-and-Reach, SAR

The European protocol [30] was used to assess flexibility of the spine and the harmstring muscles (Inter Class Coefficient = 0.98). Each participant was asked to sit with straight knees, keeping his bare feet vertically (separated by 15 cm) against a 30-cm high box (which has a ruler marked out on the upper side). The subjects, with straight knees, had to reach slowly forward over the ruler with both hands as far as possible and held the position for 2s. The distance between the vertical side of the box fingers was measured. Positive values were recorded if the participant was able to reach further than his toes (vertical side of the box), negative values were recorded if the student was unable to reach his toes as zero value was given when the participant just touched his toes.

#### 2.2.3. Cooper Endurance Run Test, CER

Also known as 12-min run; this test (reliability coefficient (ϕ = 0.96)) [31] was conducted along a 350 m-long path (in the garden of the school) where an assistant measured the *a priori* fixed distance. The students were divided in two consecutive groups running together and the total distance covered was recorded with an accuracy of 5 m.

#### 2.2.4. 30 m Sprint Test, SP-30

The participant performed a 30 m sprint (ICC = 0.96) [32,33]. The time was recorded using a chronograph (Stopwatch W073, SEIKO, Tokyo, Japan) with a time resolution of 0.01 s.

### 2.3. Statistics

Analysis of variance (ANOVA) was applied to verify differences between groups and then to evaluate changes in performance (SAR, SBJ, CER and SP-30). The analysis was carried out separately for males and females. The Bonferroni post-hoc test was applied at need to evaluate inter-years differences. The relevance of change was estimated by calculating the effect Size (ES) through calculating the Eta squared (*h*^2^), with 0.1 = small effect, 0.6 = medium effect, >0.14 = large effect [34,35]. The significance level was fixed at *p* < 0.05. All data were analyzed using SPSS version 25 (IBM-SPSS, Armonk, NY, USA).

## 3. Results

**Figure 1:**
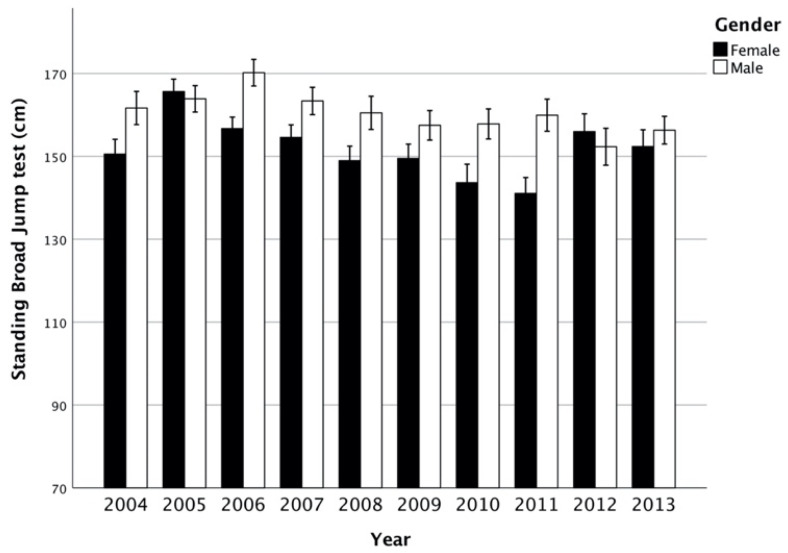
Standing broad jump test (cm; mean, interval CI 95%) between years per gender.

**Figure 2:**
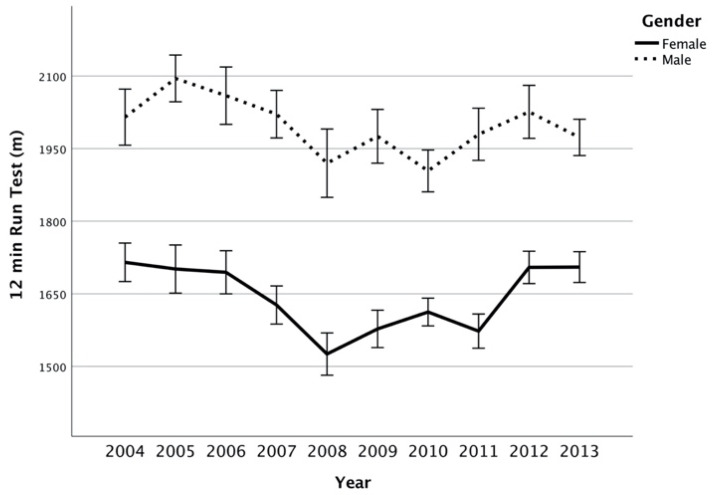
Cooper endurance run test (meters, mean, interval CI 95%) between years per gender.

**Figure 3:**
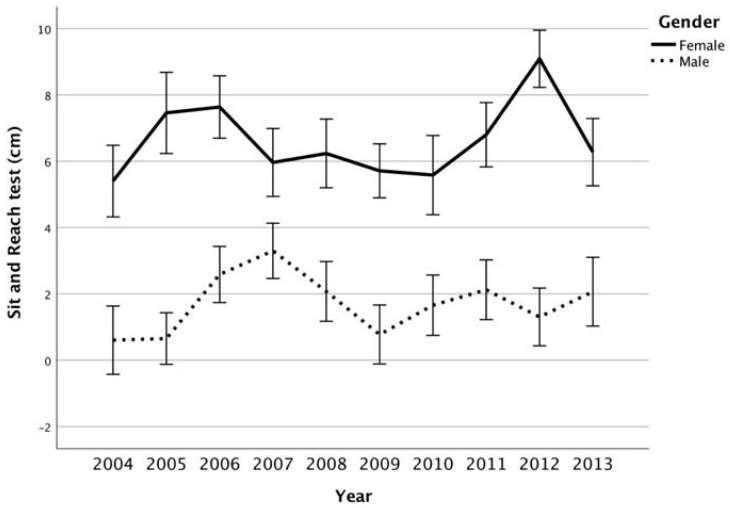
Sit-and-reach test (cm; mean, interval CI 95%) between years per gender.

**Figure 4:**
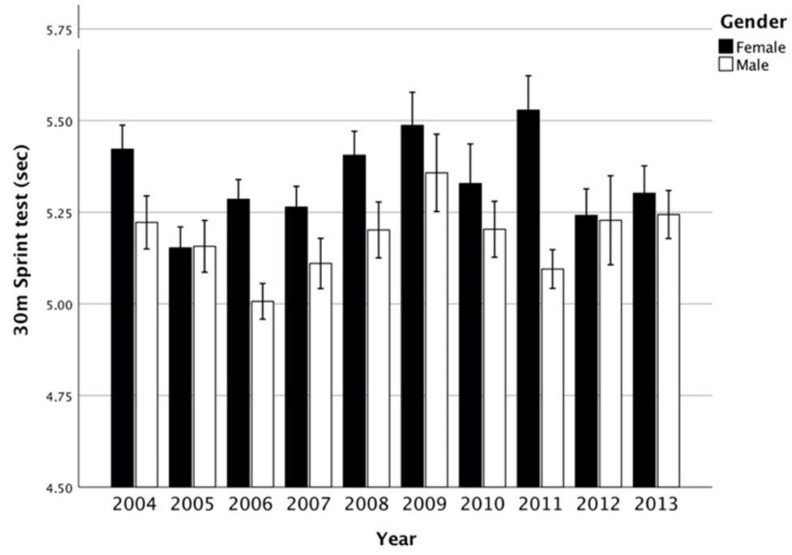
Thirty meter sprint test (seconds; mean, interval CI 95%) between years per gender.

Results of trends through the years are showed in Figure 1, Figure 2, Figure 3 and Figure 4. Trends over the years appear stable. The relevance of change expressed by *h*^2^ showed a small effect between groups, in male and female subjects (Table 2). On average, the results of SBJ were 160 cm (SD 4.89) and 152 cm (6.97) for males and females, respectively (Figure 1). Significant differences were found in males through the years (*p* = 0.042) even if the post-hoc analysis did not confirm this proof (greater difference between minimum and maximum mean values equal to 18 cm). Conversely, no difference was documented among females (*p* = 0.057). Flexibility trials (SAR) did not show differences during the years in both sexes (Table 1). Males had results between 0.7 and 2 cm, while females on average were 6.6 ± 1.1. In male groups, the CER test showed mean values between 1904 m and 2095 m in 2010 and 2005, respectively, while females had the maximum differences in mean value of 190 m (Figure 2). ANOVA test showed significant differences (Table 2) both in male and female groups throughout the years but these associations were not confirmed by Bonferroni post-hoc. In the SP-30 test, no significative differences were found (Table 2). In particular, male group performed the test in 5.18 ± 0.46 s, whereas no group performed less than 5 s or over 5.5 s (coefficient of variation = 0.09). The results in the female groups followed the same trend of male-peers but with a longer mean time of sprint (0.15 ± 0.44 s; Coefficient of Variation = 0.08).

## 4. Discussion

The present study tried to provide new insights into the physical fitness of Italian children covering physical fitness dimensions. We investigated the performance of explosive strength performances in the lower limbs (SBJ), endurance (CER), flexibility (SAR) and speed (SP-30) of sixth grade Italian students throughout a 10-year observational period (2004–2013).

While strength and speed performances remained nearly stable with a slightly positive trend over the years, the cardiorespiratory fitness revealed a continuous decline from 1970 to 2003, consistently with a global trend.

The results of this study, despite being in contrast with studies that indicate a decline in physical fitness [14,15] are in line with other research [2,36,37,38] confirming the stability of the investigated performances or even an actual improvement over the years. The following discussion is aimed at judging the extent to which the decline in physical fitness of the Italian youth can be effectively considered.

### 4.1. Standing Broad Jump, SBJ

Lovecchio et al. [11] showed an average SBJ performance of 147–155 cm in females and 165–167 in males from a northeastern Italian sample of 11–12-year-old students (female = 951; male = 505) in 2011. This trend was also confirmed by an allometric study in 2015 [39] in which, 11–12-year-old youngsters performed a similar performance. Another Italian survey (September–October 2016 that followed the preset period 2004–2013), which compared the data with other five nations [24], confirmed mean values of 150 and 160 cm, in females and males, respectively. Examining other European larges scales, a true decline cannot be unveiled based on objective data. In fact, in a meta-analysis from 1988 to 2016, including European reports on 2.5 million students (Tomkinson et al.), [12], 11-year-old adolescents jumped (50th percentile) 156 cm (males) and 144 cm (females), on average. These data were absolutely aligned with the performances investigated between 2004 and 2013. Similar result was obtained by Cohen et al. [40], who reported an increase, rather than a decline, in 11-year-old British students’ performance (SBJ) from 1998 to 2008. Other European studies showed similar findings over the years. For example, Deforche et al. [36], with the Eurofit-Barometer 1997 data, registered in Flemish young students, performances ranging from 148 to 164 cm for females and males, respectively. Interestingly, in another data collection (2004–2009) in northern Europe, Sauka et al. [38] found a jump performance of 152 cm for males and 142 cm for females: these results were slightly lower than our sample of 2004–2013 which adduced an increase over time. Moreover, Hebbelick et al. [41], reporting data from an inquiry conducted in 1993, demonstrated that the performance of young Flemish vegetarians (*n* = 82) was lower than in our sample. Also, three waves (2003–2006, 2009–2012, 2013–2017) of 11–13 years German young students confirmed steady performances [42]. Likewise, Australian normative percentiles, from 1985 to 2009, exhibited 140 and 149 cm for female and 11-year-old males (50th percentile) [43], respectively. Furthermore, data from a sample from South Africa (2010–2015) on the same test, in peer-matched children, showed similar results [44]. It is noteworthy that, among the studies of a large-scale-survey review [45], two of them reported a trend towards an increased performance of the same test, while other two studies observed no difference over a ten-year observational period. In other studies, a possible decline was supported by a 7, 9, or 11-mm difference, which is fran kly biologically irrelevant, especially if we consider the secular changes whereas the growth trend is 15 mm per decade [46]. Over time, this decline stands as harshly arguable. These values highlight a substantial steadiness: maximum value of CV = 3 and 4 in males and females, respectively.

### 4.2. Sit-and-Reach, SAR

Traditionally, the sit-and-reach test, according to the European procedure (very different than the “V-leg position” often used within the procedure proposed by ACSM) [47], has revealed poor performances globally across all investigated age groups. As previously reported, the Italian performance seems therefore to be constant over the years. In 2011 [11], found performances close to zero in males and 6.5 cm in females, which correspond to the present study results. Thereafter, an extensive data collection on more than 32,000 students aged 11–14 in six countries including Italy [24] acknowledged a stabilization of the performances, with males between 1 and 2 cm; females between 2 and 7 cm. As to this physical fitness dimension, even though in a slightly different version of it (stand and reach test), the aforementioned report [42] revealed a constant performance between 2003 and 2017. Also, within Canadian datasets (ACSM-American College of Sport Medicine protocol of SAR test) were reported, in 11–14 years old students, a substantial stables outcomes during three cycles (2007–2009, 2009–2011, 2016–2017) [48]. In the collection articles of Eberhardt et al. [45], one study from Greece revealed a positive secular trend (1992–2007) with an increase of 22% and 13% for boys and girls, respectively [45]. Other works, by reporting a 1.5 cm difference, are negligible from a physical fitness (not performance) standpoint. Worryingly, other data revealing low outcomes globally are of note [11,49].

### 4.3. Cooper Endurance Run Test, CER

The current datasets show a poor endurance level and even the most recently collected data (2017) in other Italian peers showed similar outcomes [50]. In fact, these more recent data presented an improvement: 1850 m and 2123 m for females and males, respectively. This trend is consistent with results obtained (before 1999) by Portuguese-speaking peers (Brazilian and Portuguese children) [51]. Subsequent endurance tests (1500 m and 2000 m run) [52] found that the females spent 1 s more every 40 m while males took 1 s more every 30 m covered. As to the difference between 1976 and 2001, a minimal decline could be demonstrated over a long observational period. Also, in the 1-mile run test, the average speed of Taiwanese boys was comparable to the one registered in the present study endurance test: 2.8 m/sec for males and 2.4 m/sec for females [53]. Similarly, Australian values on the 1-mile test also showed the same average speed for 11-year-olds from data regarding 1985–2009 period [43]. Furthermore, it seems that an average difference, close to 300 m, between the performances of males and females has remained unchanged over the years. Results on exhaustion tests such as 20-m shuttle run test (Eberhardt et al.) [45] specified that seven subsamples, out of 36, showed an increase in endurance performance, while within other six subsamples, a stagnation of endurance performance was observed. Furthermore, accuracy level has been improved with time and therefore possible assessment biases might render comparisons critical, so does the consistency of cut-offs. Even for this kind of physical fitness trial, an actual decline does not appear clear. One reason could be that exhaustion tests of such kind are typically used for athletes, whereas, in general health context, lower-pace tests should be more appropriate to evaluate endurance (low intensity, for a long period of time). In fact, present-study results provide a mean variability of 3–4% over a ten-year observational period.

### 4.4. 30 m Sprint Test, SP-30

For this test, even if largely used in on-field contexts, no large datasets are unfortunately available. Over ten years, the overall performances remained stable, as also confirmed by the variance analysis (coefficient of variation = 0.08). In the timeframe 2006–2007, another Italian study from Gallotta et al. [54], reported corroborating results in the performances of SP-30: 6 s for males and females (pooled data). Other studies evaluating this test (Greece 1997 to 2007; Spain, 2001 to 2007; Germany 2007–2015) indicated a general improvement in speed performance while others (Lithuania 1992–2002; Portugal 1993–2013) declared a stagnation or results [45]. In a similar sprint test (50 m), Australian age-paired children run at the same speed (5.6 vs. 5.4 m/sec), according to a meta-analysis collected between 1985 and 2009 [43]. Thus, even this physical fitness dimension seems to remain constant.

The evaluation of physical fitness is crucial to define the general health status of young people and would be important for further development of a concrete plan toward active lifestyle. Thus, evidence-based approaches should be preferred on mindset ones, especially when not performed on aligned test evaluations (i.e., 20 m shuttle run test and one-mile test or cooper endurance test). Contrary to common points of view, large reports along with this 10-year perspective study confirm a stagnation of physical fitness and not a decline. These trends were consistent across different sex and age groups. At the extreme of this spectrum, a residual improvement in the male performance progressively diminished and then stabilized. However, that one does not correspond to an actual shift from an improvement to a decline [55]. In fact, by comparing these results with recent international standard of Physical fitness dimension [12,24,27,29,38,45,55], a real decline is not evident. We are conscious that this study is limited to a peculiar period (2004–2013) but we are not able to find more recent data due to change of evaluation protocols. The major strength of the study is that short-term observational studies (i.e., focused on a yearly analysis) might be more appropriate than large-scale surveys since diverse physical fitness dimensions follow different patterns during growth [24,50,56], in which multi-age stage are envisaged (i.e., 7 to 11 years).

## 5. Conclusions

In conclusion, the authors reckon that the decline in physical fitness of the Italian youth is ostensible. It seems that performances in functional capacity still maintained in this decade. Further expanded investigations will verify the trend of physical fitness in the Italian adolescents.

According to these observations, PE teachers, public authorities and stakeholders should encourage active lifestyles, avoiding alarming tones and negative communication that are often responsible for opposite results. Youth could adhere more easily to dynamic lifestyles if they are aware of their potential, as well as pushed by positive communication.

PE teachers may use these national reference data to increase pupils’ physical fitness awareness [57] and promote healthy behaviors [58].

## Figures and Tables

**Table 1:** Numerosity of samples according to year of collection and gender.

Chronological Year	2004	2005	2006	2007	2008	2009	2010	2011	2012	2013
Ordinal Year	1	2	3	4	5	6	7	8	9	10
Boys	142	150	145	144	148	138	146	135	141	149
Girls	147	144	140	137	145	135	139	150	146	138
Total	289	294	285	281	293	273	285	285	287	287

**Table 2:** ANOVA *p*-value of test per gender (*p* < 0.05). Degree of freedom: 9.

	η	η^2^	*F*	*p*
Males				
CER (m)	0.123	0.015	4.81	0.009
SBJ (cm)	0.110	0.012	6.98	0.042
SP-30 (s)	0.101	0.010	5.80	0.103
SAR (cm)	0.098	0.010	3.78	0.131
Females				
CER (m)	0.130	0.017	12.41	0.004
SBJ (cm)	0.108	0.012	14.66	0.057
SP-30 (s)	0.106	0.011	9.48	0.070
SAR (cm)	0.077	0.006	4.99	0.502

CER: Cooper Endurance Run; SBJ: Standing Broad Jump; SP-30: 30 m Sprint test; SAR: Sit and Reach.
